# A Weibull Distribution-Based Corrosion Rate Model for Intelligent Monitoring of Steel Structures in Marine Splash Zones

**DOI:** 10.3390/s26041401

**Published:** 2026-02-23

**Authors:** Quanfeng Ouyang, Jiahuan Rao, Chuanrui Guo

**Affiliations:** 1 National Key Laboratory of Green and Long-Life Road Engineering in Extreme Environment (Shenzhen), College of Civil and Transportation Engineering, Shenzhen University, Shenzhen 518060, China; quanfengouyang7@gmail.com (Q.O.); raojiahuan0@gmail.com (J.R.); 2 Shenzhen Key Laboratory of Safety and Health Monitoring of Marine Infrastructures, Shenzhen University, Shenzhen 518060, China

**Keywords:** steel corrosion, marine splash zone, corrosion monitoring, Weibull distribution, model validation

## Abstract

Steel structures in marine splash zones (MSZ) experience severe corrosion owing to high humidity and frequent wet–dry cycles, which poses considerable threats to structural integrity and operational safety. To achieve intelligent, real-time corrosion monitoring, this study presents a corrosion-rate model based on the Weibull distribution, intended to serve as the core algorithm of smart corrosion sensors that continuously provide corrosion depth data via techniques such as electrochemical impedance spectroscopy or fiber optic sensing. The model was validated through systematic laboratory salt-spray cyclic tests that simulated MSZ conditions; corrosion behaviour was analysed by means of mass-loss measurements, electrochemical impedance spectroscopy (EIS), scanning electron microscopy (SEM) and energy-dispersive X-ray spectroscopy (EDS). The results reveal a three-stage corrosion progression and confirm that the Weibull model accurately captures the time-variant corrosion behaviour under different splash intensities. The model thus provides a reliable algorithmic foundation for intelligent corrosion monitoring, enabling real-time assessment of structural safety and prediction of residual life.

## 1. Introduction

Marine environments pose significant challenges to the longevity and safety of offshore steel structures, with corrosion being a primary and pervasive concern. This degradation process not only reduces the service life of structures but also results in substantial economic losses and increased safety risks. According to the World Corrosion Organization (WCO), global economic losses attributable to corrosion amount to approximately 3% of the world’s Gross Domestic Product (GDP) annually [1]. In China alone, corrosion-related losses reached two trillion RMB in 2014, accounting for 3.34% of the national GDP [2]. Concurrently, the global transition toward renewable energy has driven rapid expansion in offshore construction. By 2023, the cumulative installed capacity of offshore wind power had reached 75.2 GW, predominantly supported by fixed steel tubular monopile and jacket foundations [3]. Nevertheless, these steel structures remain highly vulnerable to corrosion in marine environments.

The corrosive impact of the marine environment on steel structures is commonly classified into several zones: atmospheric, splash, tidal, immersion, and seabed mud zones [4,5]. Among these, the splash zone is particularly aggressive due to continuous seawater splash and wave action, which create conditions of high humidity and frequent wet–dry cycles. These conditions accelerate electrochemical reactions between steel and corrosive agents in seawater, significantly intensifying the corrosion process. As a result, corrosion in the splash zone leads to mass loss and cross-sectional weakening of structural members, thereby reducing load-bearing capacity and compromising structural integrity. Although protective measures such as coatings are typically applied during design and construction, prolonged exposure can cause coating detachment or cracking, ultimately leading to structural degradation [6,7]. Addressing these challenges requires a thorough investigation into the corrosion behavior, mechanisms, and rate modeling of steel structures in splash zones—a research endeavor with important scientific and engineering implications. Such work is essential for improving corrosion protection strategies, refining safety assessment protocols, and enhancing lifespan prediction methods for offshore steel infrastructure.

Experimental research on marine corrosion generally follows two approaches: in situ coupon exposure in actual marine settings and laboratory-simulated tests under controlled conditions [8]. Although in situ tests can accurately reflect real-world corrosion patterns, they are often limited by long durations, high variability, and poor repeatability. Consequently, laboratory-based simulations have gained wider adoption, allowing rapid data acquisition under reproducible conditions [9]. The use of salt spray tests to simulate marine corrosion dates back to 1914, when J.A. Capp first introduced the method; it was later standardized as ASTM B117 in 1962 [10]. Subsequent studies, such as those by D.M. Drazic et al. [11], demonstrated that cyclic wet–dry salt spray testing could effectively predict long-term corrosion behavior. Further refinements were made by S.B. Lyon et al. [12], who incorporated periodic drying phases to better replicate natural exposure conditions. Despite the widespread use of cyclic wet–dry salt spray tests for simulating marine corrosion, the detailed corrosion process of steel in splash zones remains insufficiently understood, and the influence of splash intensity on corrosion progression is still unclear.

Developing accurate corrosion rate models is essential for understanding corrosion mechanisms and predicting the service life of offshore steel structures. Existing models can be broadly categorized into three types: linear [13,14], power-law [15], and statistical distribution models [16]. The linear model proposed by Southwell et al. [17] assumed a constant corrosion rate over time, but practical observations have revealed significant deviations from this assumption. To address this limitation, Melchers et al. [18] introduced a bilinear model to describe time-dependent changes in corrosion rates. Kee Paik J et al. [19] further advanced this approach by identifying three distinct corrosion phases: an initial period of effective protection, a transition phase during which protection deteriorates, and a final phase of accelerated corrosion. More recently, Wang et al. [20] advocated the use of the Weibull distribution to better capture the dynamic relationship between corrosion rate and time, demonstrating its ability to reflect time-varying corrosion behavior. However, the suitability of the Weibull distribution model for corrosion in marine splash zones, as well as its applicability under varying splash intensities, has not been established. In addition, Fiber Bragg Grating (FBG) sensors and OFDR-based distributed fiber optic sensors (OFDR-DFOS) have demonstrated significant potential in the field of corrosion rate assessment owing to their unique advantages. Xu, L. et al. [21] employed Optical Frequency Domain Reflectometry (OFDR) technology to achieve distributed strain measurements with high spatial resolution (sub-millimeter) by measuring the wavelength shift in Rayleigh backscattering in the optical fiber. This approach enables real-time capture of the corrosion process, precise localization of pitting corrosion, and accurate assessment of corrosion severity.

Therefore, this study proposes a Weibull distribution-based corrosion rate model to serve as the analytical core of smart corrosion monitoring sensors. The model is designed to convert real-time sensor data—specifically, corrosion depth measurements acquired by embedded or attached smart sensors—into assessments of structural safety and residual life, overcoming the limitations of traditional linear and power-law models in representing time-varying corrosion behavior. To validate its feasibility and superiority, a comprehensive laboratory test program was conducted under simulated marine splash zone conditions. The tests included mass loss measurements, electrochemical impedance spectroscopy (EIS), and morphological analyses (SEM/EDS), and covered a range of splash intensities. These experiments were designed to generate the data required for calibrating the model and rigorously evaluating its performance against empirically observed corrosion progression.

## 2. Materials, Specimens and Methods

This chapter describes the experimental program designed to generate data for validating the proposed Weibull-based corrosion monitoring model. The tests simulated the aggressive conditions of the marine splash zone under controlled splash intensities, providing the empirical basis required for model calibration and performance evaluation.

### 2.1. Test Materials

The steel materials commonly used in marine structures such as offshore wind turbines include grades with yield strengths of 355, 420, 460, and 500 MPa. Q355 steel was selected for this study owing to its widespread application. Its chemical composition is listed in Table 1, meets the requirements of GB/T 1591-2018 High strength low alloy structural steels [22]

### 2.2. Specimens

#### 2.2.1. Specimens for Mass-Loss Tests

**Figure 1:**
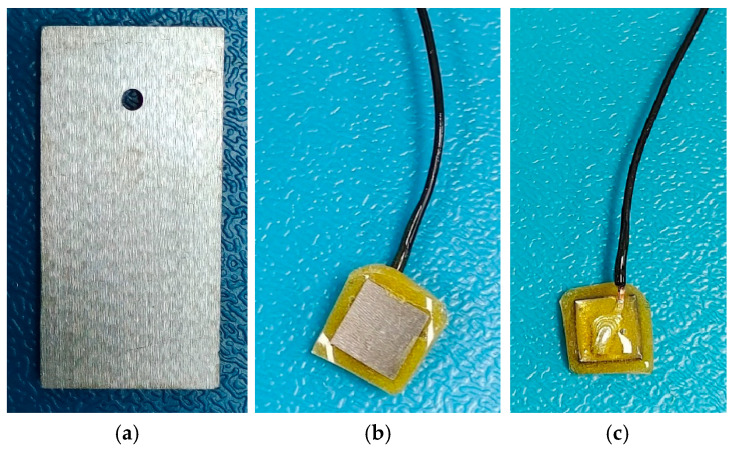
Experimental specimens: (**a**) mass loss specimen; Electrochemical analysis specimen: (**b**) working surface; (**c**) epoxy resin encapsulated surface.

All specimens were machined using computer numerical control (CNC). The mass-loss specimens were fabricated according to standard procedures [23]. Rectangular coupons measuring 50 mm × 25 mm × 5 mm were cut from Q355 steel plate. The thickness of 5 mm was selected to provide sufficient material for accurate mass loss measurements, in accordance with ASTM G1-03 [23]. A 3 mm diameter hole was drilled at one end, with its center located 10 mm from the top edge, as shown in Figure 1a. The specimens were sequentially ground and polished with 360#, 600#, 800#, and 1200# grit abrasive papers under water irrigation, followed by final polishing with W5 diamond paste to obtain a smooth, flat surface.

After polishing, the specimens were immersed in acetone to remove oily residues, rinsed thoroughly with deionized water, and then cleaned ultrasonically in anhydrous ethanol for 5 min to eliminate surface contaminants. They were dried with an air stream and subsequently placed in a preheated oven at 80 °C for 24 h. The initial mass of each specimen was measured using an electronic balance with a precision of 0.001 g.

#### 2.2.2. Specimens for Electrochemical Analysis

Specimens for EIS were prepared as 10 mm× 10 mm× 3 mm rectangles of Q355 steel. A thinner specimen (3 mm) was used to facilitate cutting and epoxy encapsulation, allowing a precise and reproducible 1 cm^2^ working electrode area. After undergoing the same polishing, degreasing, and drying procedures as the mass-loss specimens, a 2 mm diameter copper wire (10 cm in length) was soldered to one side. The opposite face was preserved as the working surface. All surfaces except the working area were encapsulated with epoxy resin to ensure insulation and sealing [24]. The working surface was then ground to remove any residual resin, resulting in an exposed electrode area of 1 cm^2^ (Figure 1b,c).

### 2.3. Salt Spray Cyclic Test

Cyclic wet–dry tests were conducted in a salt spray chamber to simulate the marine splash zone environment. Each cycle comprised 20 min of spraying followed by 10 min of drying, giving a total cycle duration of 30 min. The chamber temperature was maintained at 25 °C. This temperature was chosen to represent the typical annual average seawater temperature in temperate marine splash zones, thus avoiding the accelerated corrosion mechanisms that may occur at elevated temperatures (e.g., 35 °C specified in ISO 9227 for neutral salt spray testing) and ensuring environmentally relevant corrosion rates. Three splash intensities, represented by sedimentation rates, were investigated: 1.0–2.0, 2.0–3.0, and 4.0–5.0 mL/80 cm^2^/h, corresponding to low, medium, and high splash conditions, respectively. The corrosive medium was a 3.5 wt.% sodium chloride solution, simulating typical seawater salinity [25,26].

**Figure 2:**
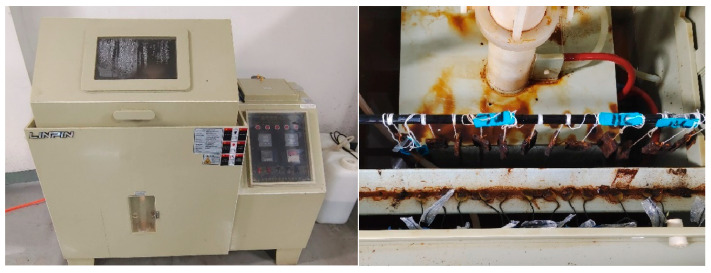
Salt spray test chamber and specimen placement.

As illustrated in Figure 2, mass-loss specimens were suspended vertically in the chamber (model LRHS-108-RY) using nylon cords. EIS specimens were placed on a rack inclined at 20° to the vertical, with a minimum spacing of 3 cm between specimens to avoid interference. This arrangement simulated key splash-zone features: ample oxygen supply, frequent wet–dry alternation, and droplet impingement.

The tests were set at 1, 2, 3, 4, 6, 8, 10, 14, 18, 22, 30, 38, 48, and 60 days, totaling 14 periods. Prior to the experiment, specimens were uniformly placed into the salt spray chamber [27]. Then the wet–dry cyclic spraying mode was initiated, with timing commencing according to the duration of the cycle. At the end of each cycle, three mass loss test specimens, three electrochemical specimens, and specimens for microscopic morphology observation were promptly removed from the salt spray chamber.

### 2.4. Characterization Methods

#### 2.4.1. Corrosion Morphology

Corroded specimens were examined using optical microscopy (OM) for an initial overview of surface damage. Selected areas were then analyzed by scanning electron microscopy (Hitachi S-3400N, Hitachi High-Tech Corporation, Mito, Japan) to reveal microstructural features such as pitting and cracking. Energy-dispersive X-ray spectroscopy (EDS, Bruker Corporation, Billerica, MA, USA) was used to determine the elemental composition of corrosion products.

#### 2.4.2. Mass Loss and Corrosion Rate Analysis

**Figure 3:**
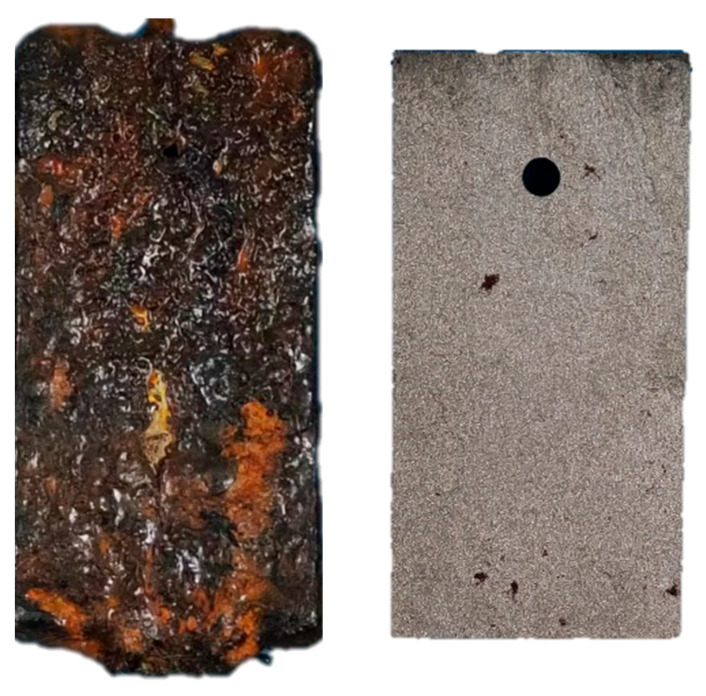
Before (**left**) and after (**right**) removal of corrosion products.

The analysis of mass loss necessitates the precise determination of specimen weight before and after corrosion exposure, requiring the meticulous removal of corrosion products from the specimens [28]. Corrosion products were removed from mass-loss specimens according to ISO 8407:2021 [28]. A cleaning solution of ammonium citrate and deionized water (1:5 by volume) was heated and held at 80 °C. After a preliminary brushing to remove loose rust, specimens were immersed in the solution for 20 min, rinsed with warm deionized water, and ultrasonically cleaned in anhydrous ethanol for 5 min. They were then dried at 80 °C for 24 h and weighed to 0.001 g precision. Figure 3 shows representative specimens before and after cleaning.

The average corrosion rate after a given exposure duration is calculated using the weight change in the specimens before and after corrosion, as shown in Equation (1). Three parallel specimens were used for each experiment, and the average mass loss from these was taken as the final data point, minimizing random errors and improving result accuracy.

(1) $$v=87,600\times \frac{m_{0}-m_{1}}{A\times T\times \rho }$$

where v represents the corrosion rate, expressed in millimeters per year (mm/a); 87,600 indicates conversion of units from hours to years and from cm to mm; $m_{0}$ denotes the pre-corrosion mass, in grams (g), measured to 0.001 g precision; $m_{1}$ denotes the post-corrosion mass, in grams (g), measured to 0.001 g precision; A is the effective corrosion surface area of the specimen, in square centimeters (cm^2^), measured to 0.01 cm^2^ precision; T is the corrosion duration, in hours (h); ρ is the specimen density, in grams per cubic centimeter (g/cm^3^). This gravimetric method follows the standard procedure described in ASTM G1-03 [23] and is widely adopted for corrosion rate determination.

The average density of Q355 steel is 7.87 g/cm^3^. While Equation (2) facilitates the calculation of the total mass consumed by corrosion of Q355 steel over time, thus determining the average corrosion rate, it does not provide precise information about the instantaneous corrosion rate. To gain deeper insights into the instantaneous corrosion behavior of Q355 steel in a simulated seawater environment, we also used the change in pre-corrosion mass and post-corrosion mass to determine the average corrosion depth, which is expressed as:

(2) $$H=\frac{m_{0}-m_{1}}{A\times \rho }$$

where H represents the average corrosion depth. Analysis of slope changes in the corrosion thickness–time curve allows for a more nuanced understanding of the instantaneous corrosion rate and its variation in Q355 steel specimens. It is worth noting that corrosion thickness refers to the difference between the specimen’s thickness before corrosion and the remaining thickness after corrosion. The specimens used for corrosion thickness and corrosion rate analysis were not protected, with all surfaces exposed to the salt mist. In contrast, the specimens used for electrochemical analysis had only one side exposed to the salt mist, as shown in Figure 1. This calculation is consistent with the mass-loss method specified in ISO 8407:2021 [29] and has been applied in previous corrosion studies.

#### 2.4.3. Electrochemical Impedance Spectroscopy

**Figure 4:**
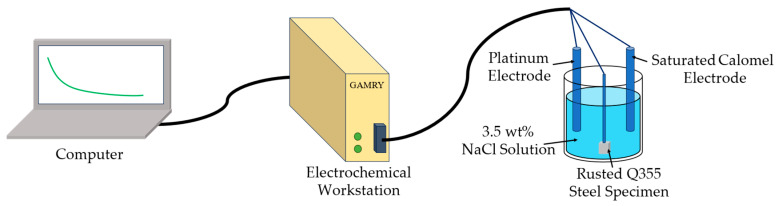
EIS experiment setup.

The EIS is conducted on a standard three-electrode workstation (GAMRY Reference 620) as illustrated in Figure 4. This three-electrode system has been widely applied in related literature [30]. The working electrode consisted of corroded specimens with varying durations, ensuring full contact between the rust-covered working surface and the simulated seawater. A saturated calomel electrode (SCE) served as the reference electrode, while a platinum (Pt) electrode functioned as the counter electrode. The experiment employed a 3.5% NaCl solution by mass fraction to simulate seawater [31]. Throughout the experiment procedure, the contact area between the working electrode’s surface and the corrosion solution was maintained at 1 cm^2^. A fixed, well-defined geometric area (1 cm^2^) is standard practice in laboratory EIS studies to facilitate reproducible calculation of current density and impedance parameters (e.g., Ω·cm^2^) [32]. While the actual electrochemically active area beneath the non-uniform rust layer may differ from this geometric area, maintaining a consistent geometric boundary condition is crucial for comparative analysis over time and across different splash intensities. The evolution of the fitted parameters (e.g., the constant phase element exponent n) inherently accounts for the changes in surface roughness and true active area.

EIS tests were performed over a frequency range of 100 kHz to 0.2 Hz, using a 10 mV amplitude sinusoidal wave signal. This frequency range was chosen to capture both the high-frequency response related to solution/interface resistance and the low-frequency response governed by charge transfer and diffusion processes typical of corroding systems with porous layers [33]. The 10 mV perturbation amplitude is a standard, widely adopted value that ensures the measurement remains within the linear response regime of the electrochemical system, as verified by preliminary tests which showed negligible variation in impedance spectra at this amplitude across different corrosion stages. Prior to conducting the EIS measurements, an open circuit potential (OCP) test was carried out to determine the steady-state corrosion potential of the system. The EIS test was then conducted at the stabilized OCP, ensuring that the system was in its natural steady state. The 10 mV amplitude perturbation voltage was applied relative to this OCP value, allowing the impedance data to reflect the intrinsic electrochemical properties of the system under stable corrosion conditions. The resulting test data were subsequently subjected to curve fitting and analysis using ZsimpWin (3.21) software.

The systematic experimental framework described in this section—encompassing specimen preparation, controlled cyclic testing, and multi-faceted characterization—provides the essential and high-quality dataset required for the subsequent calibration of the Weibull model parameters and, crucially, for the rigorous validation of its predictive accuracy against empirical corrosion progression under varying splash intensities.

## 3. Results and Corrosion Process Analysis

This section presents the key experimental evidence used to validate the proposed Weibull model. The analysis of corrosion morphology, mass-loss kinetics, and electrochemical behavior under simulated splash conditions reveals the characteristic time-dependent progression of corrosion. The identified stages and mechanisms provide the empirical foundation for evaluating the descriptive and predictive capabilities of the Weibull model in Section 4.

### 3.1. Corrosion Morphology Analysis

#### 3.1.1. Macroscopic Morphology Analysis

**Figure 5:**
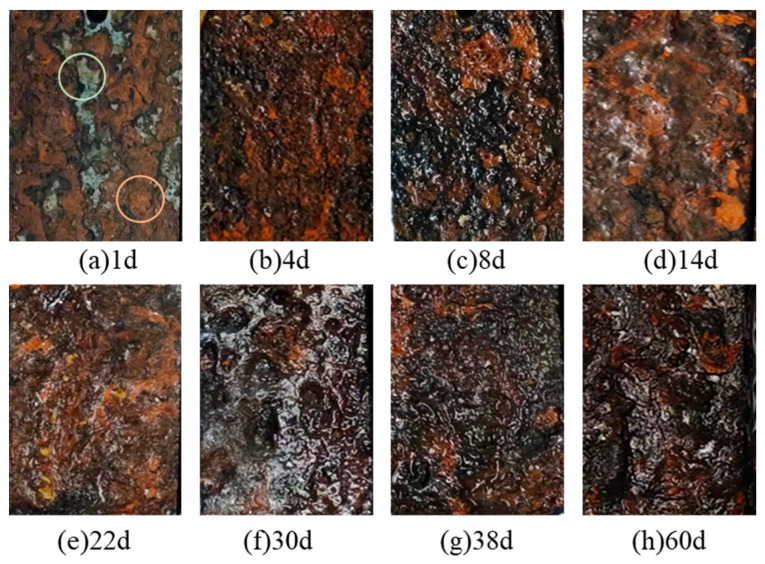
Magnified 3× image of Q355 steel surfaces after varying durations of corrosion at a sedimentation rate of 1.0–2.0 mL/80 cm^2^/h ((**a**): 1 day; (**b**): 4 days; (**c**): 8 days; (**d**): 14 days; (**e**): 22 days; (**f**): 30 days; (**g**): 38 days; (**h**): 60 days). All images share the same magnification; scale bar = 5 mm (applicable to all subfigures).

The macroscopic morphology of Q355 steel surfaces subjected to various corrosion durations under a splash environment with a sedimentation rate of 1.0–2.0 mL/80 cm^2^/h is presented in Figure 5. After one day of salt spray cyclic corrosion, as shown in Figure 5a, corrosion in the splash zone initiates primarily with pitting. Following the wet–dry spray cycles, simulated seawater sprayed onto the specimen surface to condenses into droplets, leading to localized corrosion at points of droplets contact, thus resulting in pitting. At this stage, the corrosion on the specimen surface is characterized by an uneven distribution of light green and pale-yellow rust spots, as show as the circles in Figure 5a, along with a thin layer of corrosion products.

Figure 5a,b illustrates the changes in the macroscopic morphology of Q355 steel during the rapid corrosion phase (0 d–4 d) within a simulated marine splash zone. As corrosion progresses, the rust layer on the steel surface becomes denser, with a gradual accumulation of corrosion products that coalesce previously isolated rust patches into larger areas. The color of these corrosion products transitions from pale yellow to orange and eventually to dark brown, with some black corrosion products also appearing. During this phase, the accumulation of corrosion products gradually reduces the effective contact area between the steel surface and the corrosive medium, resulting in a marked decrease in the corrosion rate.

As corrosion progressed beyond 4 days of exposure (Figure 5c–f), the rust layer continued to thicken, and the surface gradually smoothened. The color of the corrosion products deepened to black and dark brown, indicating an increased presence of stable iron oxides such as magnetite. Consequently, the corrosion rate of Q355 steel decreased steadily during this period. As the number of wet–dry cycles increases, the rust layer continues to thicken, and the surface gradually smoothens. The color of the corrosion products deepens to black and dark brown, indicating an increased presence of stable iron oxides such as magnetite. Consequently, the corrosion rate of Q355 steel decreases steadily during this period.

Figure 5g,h illustrates the stable corrosion phase (30 d–60 d), where significant thickening of the rust layer is observed, along with the appearance of more pits and holes, leading to a rougher surface texture. In addition, it is observed that the corrosion rate stabilizes at this phase. This qualitative description of the macroscopic evolution—from isolated pitting to full coverage and eventual roughening—provides a visual correlation with the quantitatively defined corrosion stages (rapid, slow, stable) that are rigorously demarcated by the mass-loss and corrosion rate kinetics presented in Section 3.2. Future studies employing techniques such as 3D surface profilometry or digital image analysis could yield quantitative metrics (e.g., pitting density, surface roughness Ra) to further corroborate this staged progression.

#### 3.1.2. Microscopic Morphology Analysis

**Figure 6:**
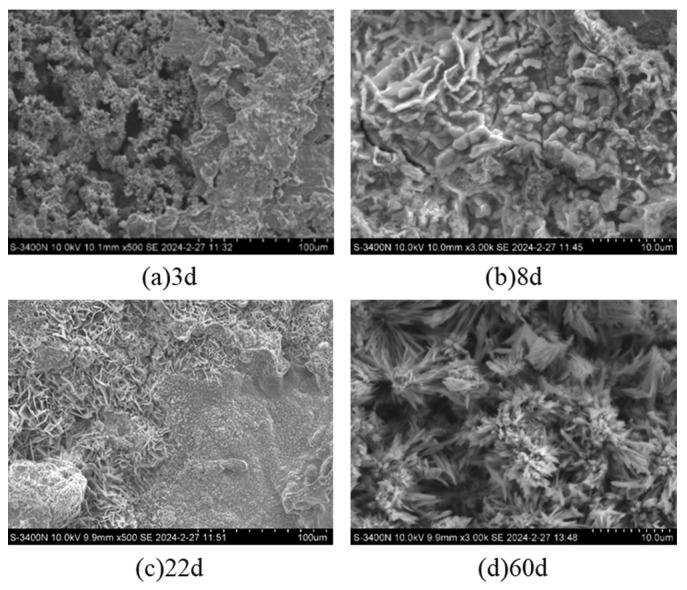
SEM images of steel corrosion morphology after various durations of corrosion.

To elucidate the corrosion mechanisms of Q355 steel, a comprehensive microscopic morphology analysis of the corrosion products was conducted at various exposure intervals. The rust layer morphology was examined using a Hitachi S-3400N scanning electron microscope after subjecting Q355 steel samples to a simulated seawater splash zone environment with a controlled sedimentation rate of 1.0–2.0 mL/80 cm^2^/h. SEM was utilized to analyze the microstructure of the corrosion products. Figure 6 displays representative SEM micrographs depicting the temporal evolution of corrosion morphology on Q355 steel under the simulated marine splash conditions.

**Figure 7:**
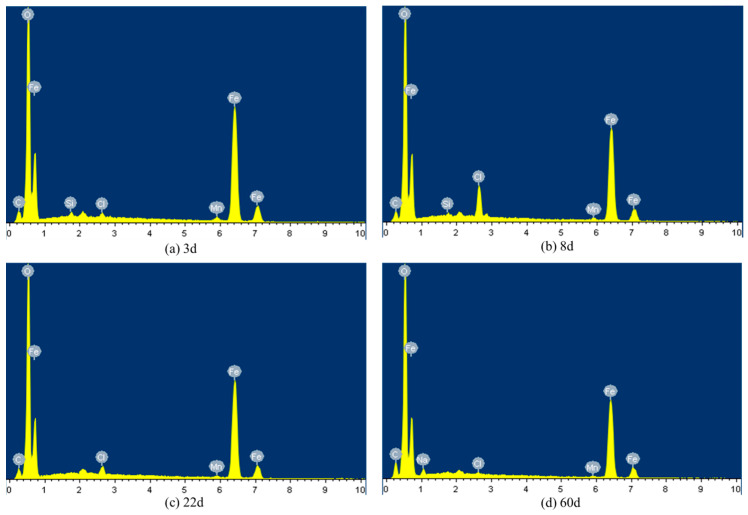
EDS analysis images of Q355 steel after various durations of corrosion.

To further elucidate the corrosion process, EDS analysis was performed to characterize the elemental composition of the corrosion products. Figure 7 displays the EDS elemental analysis spectra for Q355 steel specimens after varying exposure durations in the simulated seawater splash environment. To quantitative results of the EDS analysis, detailing the elemental composition of corrosion products on Q355 steel as a function of exposure time in the simulated marine splash environment, are summarized in Table 2. The presence of Si is attributed to its role as a common alloying element in Q355 steel (Table 1), which may be incorporated into the corrosion product layer or originate from surface impurities. The decrease in Si content from 0.405 at% (3 d) to being undetectable (60 d) suggests that silicon-containing phases (e.g., silicate or silica) may be initially present but are either diluted by the massive formation of iron oxides or are not a stable component of the mature rust layer under the given conditions. This evolution aligns with the observed densification and stabilization of the rust layer, where the dominant stable iron oxides (e.g., Fe_3_O_4_, α/γ-FeOOH) dictate the protective properties [34,35].

The morphological and compositional evolution of corrosion products on Q355 steel was examined over a 60-day period in a corrosive environment. As seen in Figure 6, after 3 days of exposure, the steel surface exhibits an uneven distribution of corrosion products, characterized by fragmented layers and flake-like structures with varying depths. The inner rust layer consisted of loosely packed, small flake-like crystalline accumulations interspersed with numerous pores.

As corrosion progressed to 8 days, the steel surface became fully covered with corrosion products, transitioning from flocculent to a combination of flocculent and short rod-like structures. The flocculent morphology is commonly associated with less crystalline, hydrated iron oxyhydroxides like lepidocrocite (γ-FeOOH), while the development of short rod-like or needle-like features in later stages (Figure 6 and Figure 7, 60 d) is often indicative of the presence of more stable phases such as goethite (α-FeOOH) or akaganéite (β-FeOOH, favored by Cl^−^) [36]. The corrosion layer demonstrated increased density, with a reduction in porosity and the emergence of partial cracks, indicative of potential delamination. Elemental composition remained similar to the 3-day sample, with a notable increase in Cl content, suggesting ongoing active corrosion. After 22 days, localized corrosion was evidenced by the appearance of larger corrosion pits and dissolution cavities. The corrosion surface exhibited a heterogeneous topography with depressions and pores, while the interior of the corrosion products displayed a complex morphology of flake-like, flocculent, and needle-like formations. Elemental analysis revealed the presence of sodium (Na) in addition to previously observed elements. A decrease in Cl content compared to the 8-day sample suggested a reduction in corrosion rate, marking the transition to a slow corrosion phase.

After 60 days of exposure, the corrosion products on Q355 steel demonstrated significant densification, with localized thickening forming clumpy and blocky structures. The surface was characterized by dense flocculent material and needle-like crystals, attributed to the deposition and crystalline growth of corrosion products. Elemental analysis showed a continued predominance of Fe and O, with detectable levels of Na, Cl, and Mn. The notable decrease in Cl content and absence of Si, compared to earlier time points, indicated the formation of dense corrosion products and a lower corrosion rate, signifying the attainment of a stable corrosion phase.

The observed evolution from localized pitting to the formation of a dense, stable rust layer corroborates a transition in corrosion mechanisms over time. This temporal evolution in surface morphology and composition highlights the non-linear, time-variant nature of the corrosion process, a key behavioral pattern that the proposed Weibull model is designed to characterize.

### 3.2. Analysis of Mass Loss Test Results

**Figure 8:**
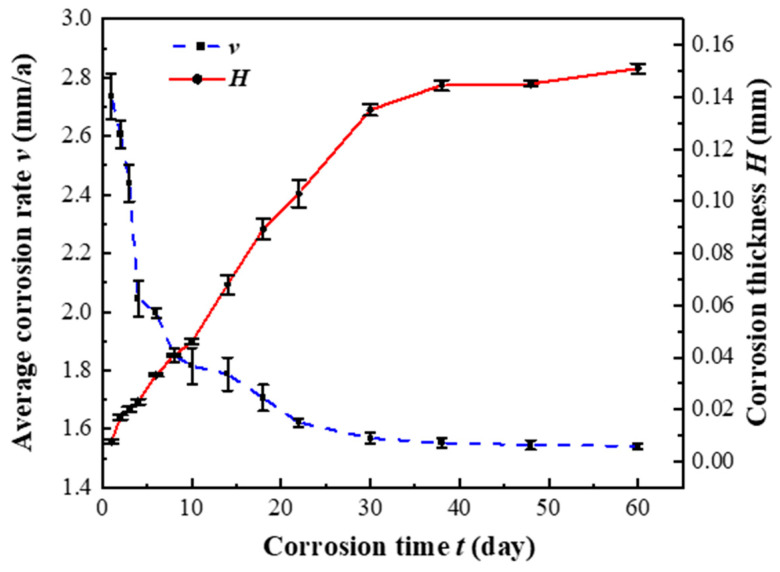
Variation curves of corrosion rate and average corrosion depth over time for Q355 steel.

Mass loss tests were conducted on Q355 steel specimens subjected to simulated splash conditions with a sedimentation rate of 1.0–2.0 mL/80 cm^2^/h. Figure 8 presents the temporal variations in corrosion rate and average corrosion depth for Q355 steel in the simulated seawater environment. The data reveal an inverse relationship between corrosion rate and time, while average corrosion depth exhibits a positive correlation with exposure duration. Based on the observed trends, the corrosion process can be delineated into three distinct phases: rapid corrosion phase (0 d–4 d), slow corrosion phase (4 d–30 d), and stable phase (30–60 d), where d denotes days of exposure.

During the rapid corrosion phase (0 d–4 d), the initial high corrosion rate rapidly decreases. This phenomenon can be attributed to the full exposure of the fresh metal surface to simulated seawater, allowing for rapid initial corrosion. The availability of active sites for oxygen reduction and chloride ion interaction promotes an accelerated corrosion rate during this phase. As corrosion products begin to form and cover the surface, they reduce the effective reaction area, leading to a decline in corrosion rate.

During the slow corrosion phase (4 d–30 d), the specimen surface becomes increasingly covered by a thickening rust layer. This layer impedes the penetration of dissolved oxygen, chloride ions, and atmospheric oxygen to the metal surface, decelerating the corrosion reaction and gradually stabilizing the corrosion rate.

In the stable corrosion phase (30 d–60 d), the corrosion products on the specimen surface reach a steady state, enhancing the specimen’s resistance to penetration by dissolved oxygen, chloride ions from the simulated seawater, and atmospheric oxygen. The progressive formation of the rust layer prevents direct contact between the corrosion medium and the metal surface, significantly limiting further corrosion. Consequently, the corrosion rate continues to decrease slowly until it stabilizes, with the rust layer playing a crucial protective role in mitigating metal corrosion.

The clear demarcation of the corrosion process into rapid, slow, and stable phases, as quantified by mass loss and corrosion rate, provides a critical empirical benchmark. The Weibull model, with its flexible shape parameter, is inherently suited to capture such distinct sequential transitions in degradation rate, offering a significant advantage over simpler models that assume monotonic trends.

### 3.3. Electrochemical Impedance Spectroscopy

**Figure 9:**
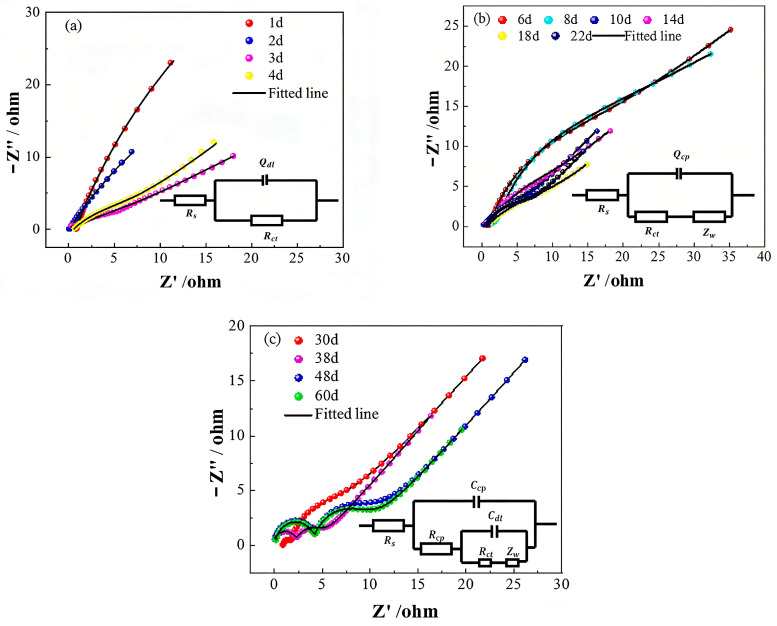
Nyquist plot of rusted Q355 steel specimens in a simulated marine splash zone.

Nyquist plots obtained from Q355 steel subjected to various durations of corrosion in a cyclic wet–dry simulated splash environment are displayed in Figure 9. By analyzing these plots, three equivalent circuit models for Q355 steel at different stages of corrosion were established.

Figure 9a shows the Nyquist plot and corresponding equivalent circuit model for the early stages of corrosion (1 d–4 d). In this model, *R*_s_ denotes the electrolyte resistance, $R_{ct}$ represents the charge transfer resistance at the interface between the Q355 steel and the sodium chloride solution, and $Q_{dl}$ denotes the constant phase element characterizing the interfacial behavior. To account for the influence of surface roughness on the working electrode, a constant phase element $Q_{dl}$ replaces the ideal capacitive component, representing the non-ideal charge transfer capacitance at the metal-solution interface. Initially, the impedance spectra exhibit an incomplete semicircle that diminishes in diameter as corrosion progresses, indicating an accelerated corrosion rate. This evolution culminates in a small semicircle in the high-frequency region and a radial line in the low-frequency region, which corresponds to a rapid decrease in $R_{ct}$. These observations suggest that the accumulation of corrosion products significantly affects mass transfer during the early stage of corrosion.

Figure 9b presents the Nyquist plot and the corresponding equivalent circuit model for the mid-stage of corrosion (5 d–22 d). In this model, $R_{ct}$ and $Q_{dl}$ represent the charge transfer resistance and constant phase element at the interface, while $Z_{w}$ denotes the Warburg impedance. The presence of a capacitive arc at mid to high frequencies, formed by $R_{ct}$ and $Q_{dl}$, reflects the diffusion of corrosion products away from the working electrode surface and the concurrent diffusion of oxygen from the solution toward the surface. This process generates the Warburg impedance in the low-frequency region, indicating a deceleration in the electrochemical charge transfer process. As corrosion progresses, a protective film of corrosion products forms on the Q355 steel surface, which moderately inhibits further corrosion, aligning with the identified slow corrosion phase.

Figure 9c shows the Nyquist plot and equivalent circuit model for the later stages of corrosion (30 d–60 d). In this model, $R_{cp}$ and $C_{cp}$ represent the charge transfer resistance and capacitance at the Q355 steel interface with the sodium chloride solution, while $R_{ct}$ and $C_{dl}$ represent the charge transfer resistance and capacitance at the interface of the corrosion products on the Q355 steel surface. The Nyquist plot for this stage consists of a semicircle in the high-frequency region and a diffusive-like semicircle with a ray in the low-frequency region. The shape of the impedance spectrum and the equivalent circuit model for this stage differ significantly from the mid-stage (Figure 9b), reflecting the development of a more complex corrosion product layer. The slight increase in the diameter of the high-frequency semicircle before stabilization indicates that the corrosion products formed in this phase significantly inhibit further corrosion, consistent with the observed stable corrosion phase.

The selection of these specific equivalent circuit models was guided by the distinct shapes of the Nyquist plots at each stage and established physical models for corroding interfaces. For the early stage (1 d–4 d), the depressed capacitive semicircle (Figure 9a) is characteristic of a charge-transfer controlled process at a rough or inhomogeneous electrode surface, aptly modeled by a parallel RQ (Resistor-Constant Phase Element) combination. The emergence of a ~45° Warburg tail in the mid-stage Nyquist plots (Figure 9b) is a definitive signature of diffusion-limited kinetics, necessitating the addition of a Warburg element (W) to the circuit. Finally, the appearance of two time constants in the later stage (Figure 9c)—a depressed semicircle at high frequency and a diffusion-influenced feature at low frequency—reflects the formation of a dual-layer structure: a dense inner rust layer and a porous outer layer. This is commonly represented by a nested (R(C(R(Q(RW))))) type circuit, where distinct R-C/R-Q pairs model the different layers [37].

These three models were selected to reflect the changing corrosion mechanisms at different stages of exposure. In the early stages, corrosion is dominated by charge transfer at the metal-solution interface. As corrosion progresses, the presence of corrosion products requires the inclusion of Warburg impedance to represent diffusion processes. In the later stages, additional elements are incorporated to represent the layered structure of corrosion products, which effectively hinder further corrosion. Table 3, Table 4 and Table 5 show the curve-fitting results of each model.

**Figure 10:**
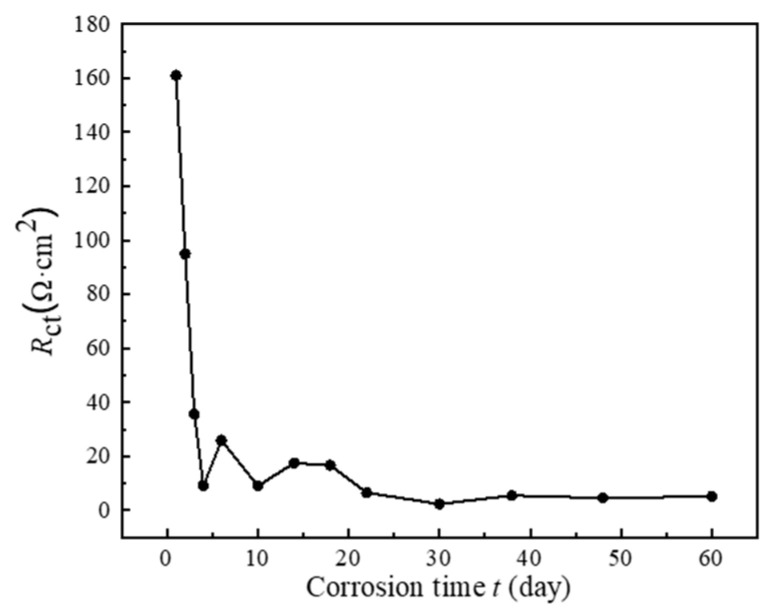
Fitted $R_{ct}$ values from EIS data for rusted Q355 steel specimens in a simulated marine splash zone.

The impedance value $R_{ct}$ is typically inversely correlated with the corrosion rate [32]. Figure 10 illustrates a decrease in $R_{ct}$ across different corrosion durations, with a notable minimum at day 60. This general decrease in $R_{ct}$ initially aligns with an increase in corrosion rate, evidenced by mass loss data. However, the correlation diverges during the mid to late stages of the experiment, where $R_{ct}$ levels off and then slightly increases, suggesting the formation of a more protective corrosion layer which may not effectively reduce the mass loss. While the charge transfer resistance ($R_{ct}$) decreases rapidly in the early stages of corrosion, the mass loss continues to increase progressively over time. This behavior is expected as $R_{ct}$ reflects the resistance to electrochemical reactions at the metal-solution interface, and its decrease suggests an increase in corrosion activity, especially during the initial stages. Mass loss, on the other hand, is a cumulative measure of material degradation, which continues even as $R_{ct}$ stabilizes or remains low. Therefore, despite the seemingly opposing trends, $R_{ct}$ and mass loss describe different aspects of the corrosion process, with $R_{ct}$ highlighting electrochemical reactivity and mass loss reflecting overall material loss.

This divergence prompts a more nuanced discussion of the corrosion process, where initial rapid metal dissolution is gradually hindered by the development of a corrosion product layer. The protective quality of this layer may vary, reflecting differences in $R_{ct}$ and mass loss trends. It’s possible that during the mid-stages of corrosion, the layer’s protective properties were compromised, perhaps due to environmental factors or changes in the layer’s morphology, before stabilizing towards the end of the testing period.

The evolution of equivalent circuit models and the fitted parameters (e.g., $R_{ct}$) across the three corrosion stages reflects the changing interfacial properties and mass transfer limitations over time. These electrochemical signatures of time-varying corrosion behavior provide an independent set of data against which the kinetic trends predicted by the Weibull model can be cross-validated.

The evolution of the equivalent circuit models, from a simple R(QR) to R(QR)(W) and finally to R(C(R(C(R(W))))) circuits, aligns with the progressive development of a heterogeneous and stratified rust layer. The increasing complexity of the interfacial structure, as reflected in the circuit models, is consistent with the reported behavior of steel under cyclic wet–dry conditions where the corrosion product layer’s resistance and diffusion properties become dominant factors over time [38]. The use of Constant Phase Elements (CPEs) instead of ideal capacitors is critical for accurately modeling the non-ideal capacitive behavior arising from surface roughness, porosity, and inhomogeneity of the rust layer, a well-established practice in modern EIS analysis of corroding metals [39]

## 4. Development and Validation of a Weibull-Based Corrosion Monitoring Model

Building on the systematic analysis in Section 3, which revealed a triphasic corrosion evolution (rapid, slow, and stable stages) accompanied by transformations in rust layer morphology and electrochemical mechanisms (as evidenced by the evolving EIS spectra and equivalent circuits), this study formally proposes a corrosion depth prediction model based on the Weibull distribution. The model serves as the core analytical algorithm for intelligent corrosion monitoring sensors, designed to translate real-time sensor data into quantitative assessments of structural corrosion status and residual life. This approach overcomes the inherent limitations of traditional linear or power-law models in describing dynamic corrosion processes.

### 4.1. Proposal of the Weibull Corrosion Monitoring Model

The corrosion rate can be described by a Weibull distribution function, often referred to as the Weibull corrosion model [32]. The corrosion rate formula is given as:

(3) $$r\left(t\right)=\left\{\begin{array}{ll} \;\;\;\;\;\;\;0 & 0\leq t\leq T_{st}\\ d_{\infty }\frac{\beta }{\alpha }{\left(\frac{t-T_{st}}{\alpha }\right)}^{\beta -1}exp\left\{-{\left(\frac{t-T_{st}}{\alpha }\right)}^{\beta }\right\} & T_{st}\leq t\leq T_{L} \end{array}\right.$$

where $d_{\infty }$ represents the maximum value of the average corrosion depth, β is the shape parameter, α is the scale parameter, and $T_{st}$ is the location parameter, which corresponds to the time at which corrosion begins. The time at which the corrosion rate reaches its maximum value can be calculated by

(4) $$T_{A}=\left\{\begin{array}{ll} T_{CL}=T_{st}+\alpha {\left(\frac{\beta -1}{\beta }\right)}^{\frac{1}{\beta }} & \beta >1\\ \;\;\;\;T_{st} & \beta \leq 1 \end{array}\right.$$

while the maximum corrosion rate can be calculated by

(5) $$r_{max}=\left\{\begin{array}{ll} d_{\infty }\frac{\beta }{\alpha }{\left(\frac{\beta -1}{\beta }\right)}^{\frac{\beta -1}{\beta }}exp\left(\frac{1-\beta }{\beta }\right) & \beta >1\\ \;\;\;\;\;d_{\infty }\frac{\beta }{\alpha } & \beta =0\\ \;\;\;\;\;\infty & \beta >1 \end{array}\right.$$

The Weibull model exhibits excellent adaptability to different corrosion environments and can accurately calculate the corrosion thickness at any given time, which is expressed as

(6) $$d\left(t\right)=\left\{\begin{array}{ll} \;\;\;\;\;0 & 0\leq t\leq T_{st}\\ d_{\infty }\left\{1-exp\left[-{\left(\frac{t-T_{st}}{\alpha }\right)}^{\beta }\right]\right. & T_{st}\leq t\leq T_{L} \end{array}\right.$$

For corrosion specimens that have not undergone any corrosion protection treatment, such as the bare steel used, corrosion phenomena become evident within a very short exposure time. For such components lacking corrosion protection measures, the corrosion initiation time can be assumed as $T_{st}=0$, and the corrosion model can be expressed as

(7) $$\;d\left(t\right)=d_{\infty }\left\{1-exp\left[-{\left(\frac{t}{\alpha }\right)}^{\beta }\right]\right.$$

Parameter estimation for the Weibull distribution has been extensively studied. Common methods include the method of moments, linear regression, and maximum likelihood estimation (MLE), with MLE being particularly effective for the two-parameter form [33,34]. In this work, the model is reduced to a two-parameter formulation (β, α) by fixing $T_{st}=0$. The parameter $d_{\infty }$ is initialized based on the measured average corrosion depth. Subsequently, the shape parameter β and scale parameter α are estimated using the maximum likelihood method.

### 4.2. Validation and Comparative Analysis Using Experimental Data

This section calibrates and validates the proposed Weibull model using the experimental data obtained under a specific splash intensity (sedimentation rate: 1.0–2.0 mL/80 cm^2^/h), as detailed in Section 3. A comparative analysis against conventional linear and power-law (exponential) models is conducted to demonstrate the superior capability of the Weibull model in characterizing the time-dependent corrosion process.

**Figure 11:**
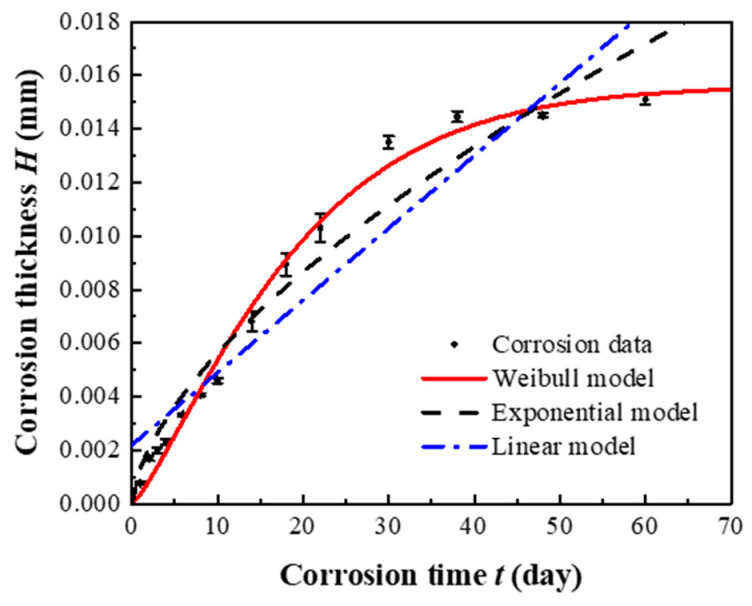
Comparison chart of fitted curves for average corrosion depth of Q355 steel.

The average corrosion depth of Q355 steel under the specified splash intensity was calculated using the mass-loss data. The experimental data were fitted using three models: a linear model, an exponential (power-law) model, and the proposed Weibull model. Figure 11 presents a comparative plot of the fitted curves against the experimental data points, and the corresponding functional expressions are listed in Table 6.

To rigorously validate the performance of the proposed model, its fitting results were compared with those of a linear model and a traditional power-law (exponential) model applied to the same dataset. Figure 11 visually compares the fitted curves of all three models against the experimental data points, while Table 7 summarizes the results of multiple statistical goodness-of-fit tests.

The analysis leads to the following conclusions:

Superior Explanatory Power: The Weibull model achieves the highest coefficient of determination ($R^{2}=0.9913$), significantly outperforming the linear ($R^{2}=0.8889$) and exponential ($R^{2}=0.9522$) models, indicating it explains the greatest proportion of variance in the experimental data.

Best Agreement with Measurements: The Weibull model exhibits the most favorable statistical metrics, notably the lowest χ^2^/DoF value (0.1818), demonstrating minimal discrepancy between predictions and measurements—a critical requirement for a reliable monitoring algorithm.

Accurate Capture of Nonlinear Behavior: Visually, only the Weibull model accurately follows the curvature of the data throughout the entire exposure period (Figure 11). It successfully captures the non-linear transition between the identified corrosion phases (rapid → slow → stable), whereas the linear model fails to represent any curvature, and the exponential model shows systematic deviation in the mid-to-late stages.

This comprehensive validation confirms that the Weibull model provides a robust and accurate mathematical representation of the time-varying corrosion progression, forming a solid foundation for its role as the core algorithm in intelligent corrosion monitoring systems.

### 4.3. Interpretation of Model Parameters and Corrosion Mechanisms

For a mathematical model to transition from a curve-fitting tool to a reliable engine for intelligent monitoring, the physical interpretability of its parameters is paramount. This section establishes clear links between the Weibull model parameters and the underlying corrosion processes, enhancing the model’s credibility and providing actionable insights for diagnostic monitoring.

**Figure 12:**
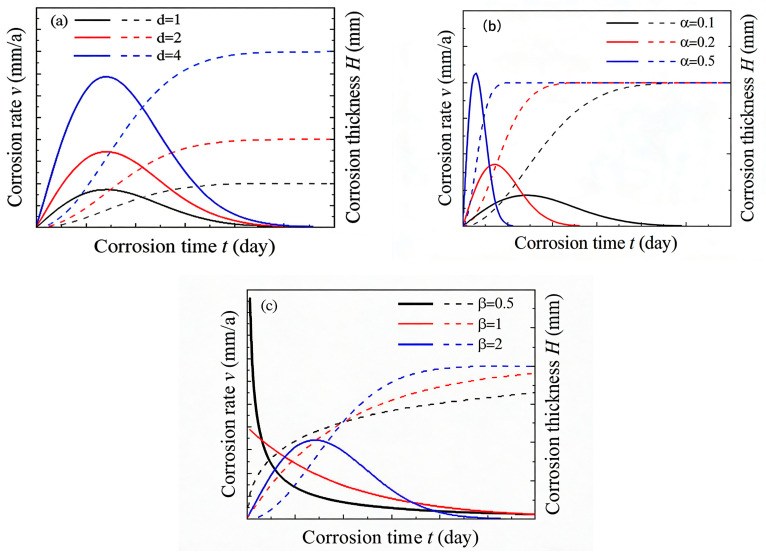
Impact of parameter variations on the corrosion model: (**a**) Upper limit of corrosion depth $d_{\infty }$, (**b**) Scale parameter α, (**c**) Shape parameter β.

The influence of key parameters on the corrosion depth curve is illustrated in Figure 12:

Maximum Corrosion Depth ($d_{\infty }$): Governs the long-term corrosion potential or saturation level (Figure 12a).

Scale Parameter (α): Acts as a characteristic time constant; a smaller α indicates faster progression to the characteristic corrosion state (Figure 12b). The condition α > 1 (as fitted in this study) mathematically ensures that the corrosion rate function r(t) in Equation (3) has a single maximum (when β > 1), corresponding to the peak rate observed in the rapid phase. Physically, this reflects a kinetic transition. In the early stage, corrosion is primarily interface-controlled, with rate limited by the charge transfer reaction at the bare metal surface. As the rust layer grows, the transport of reactive species (e.g., O_2_, Cl^−^) through this layer becomes rate-limiting, shifting the process to diffusion control. The Weibull model captures this transition through its shape; the parameter α scales the time at which this shift becomes dominant, linking it to the rust layer’s evolving protective property [20].

Shape Parameter (β): Controls the shape of the rate–time curve. A value β > 1 produces a rate that increases to a maximum before decreasing, which aligns with the observed corrosion phases (Figure 12c).

The fitted parameters thus become quantitative descriptors of the corrosion narrative:

The convergence toward a stable $d_{\infty }$ is consistent with the formation of a dense, stable rust layer (observed via SEM/EDS at 60 days) that significantly retards further corrosion.

The fitted α > 1 validates that the model inherently captures the shift in corrosion mechanism from an interface-controlled reaction (early stage) to a diffusion-controlled process through a rust layer (later stage).

The value of β is influenced by factors controlling early-to-mid-term kinetics, such as the frequency of wet–dry cycles (splash intensity), which govern electrolyte renewal and oxygen supply.

In summary, the proposed Weibull model transcends being a high-accuracy fitting function. Its parameters ($d_{\infty }$, α, β) are imbued with clear physical and mechanistic meanings related to long-term damage limits, environmental severity, and the kinetic mode of degradation, respectively. This interpretability is a profound advantage for intelligent monitoring. By tracking these fitted parameters over time, a monitoring system can not only predict future corrosion depth but also diagnose the current corrosion stage and infer the dominant underlying mechanism, transforming the sensor system into a truly insightful prognostic and health management tool.

## 5. Validation of Model Adaptability to Varying Splash Intensities

To rigorously evaluate the adaptability of the proposed corrosion model and to understand its performance and parametric variation under different environmental conditions, tests were conducted under simulated splash zones with varying intensities. The splash intensity was controlled by adjusting the sedimentation rate in the salt spray chamber. Three levels were defined: low (condition *a*: 1.0–2.0 mL/80 cm^2^/h), medium (condition *b*: 2.0–3.0 mL/80 cm^2^/h), and high (condition *c*: 4.0–5.0 mL/80 cm^2^/h). This setup allowed for observing the model’s response and parameter evolution across a range of splash severities.

### 5.1. Electrochemical Behavior as Corroborative Evidence for Model Universality

**Figure 13:**
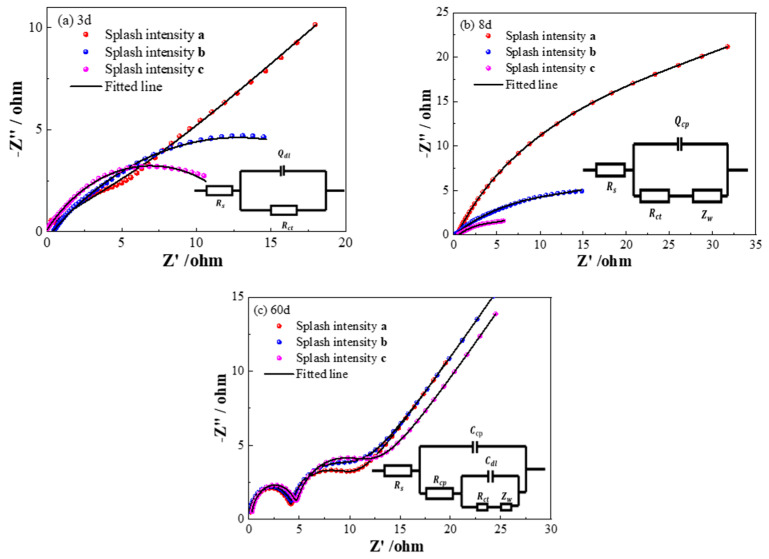
Nyquist plots of Q355 steel samples with rust layers under different splash intensities.

EIS provides an independent, mechanistic perspective on the corrosion process. Figure 13 presents the Nyquist plots and corresponding equivalent circuit models for Q355 steel under low (*a*), medium (*b*), and high (*c*) splash intensities.

A key finding is the consistent observation of the three-stage corrosion progression (rapid → slow → stable) across all splash intensities. The evolution of the equivalent circuit models follows the same sequence identified under a single intensity in Section 3: from a simple charge-transfer dominated model in the early stage, to a model incorporating Warburg impedance (reflecting diffusion control) in the mid-stage, and finally to a more complex model representing a stratified rust layer in the late stage. This demonstrates that the temporal evolution of the corrosion mechanism is a universal characteristic of splash zone corrosion, independent of the specific splash intensity. While the absolute corrosion rate (reflected in the size of the capacitive arcs) varies with intensity, the fundamental sequential pattern of mechanistic stages remains invariant. This universality strongly supports the application of the same Weibull model framework under different environmental severities; the shape parameter β effectively captures this common phased behavior, while the other parameters ($d_{\infty }$, α) scale the process in magnitude and time. To further substantiate this, we compared key EIS parameters across splash intensities. The *R_ct_* at the end of the rapid corrosion phase (e.g., day 4) systematically decreased with increasing splash intensity (e.g., ~9.2, ~7.5, ~5.8 Ω·cm^2^ for low, medium, high, respectively), quantitatively reflecting the higher initial corrosion activity under more aggressive conditions. Similarly, the *Y_w_*, indicative of diffusion difficulty, also showed a consistent trend of lower values (faster effective diffusion) at higher splash intensities during the mid-stage. This quantitative agreement in parameter trends across different environmental severities reinforces the conclusion that the fundamental, three-stage corrosion mechanism is universal, with kinetics modulated by splash intensity.

### 5.2. Analysis of Parameter Stability and Physical Consistency

**Figure 14:**
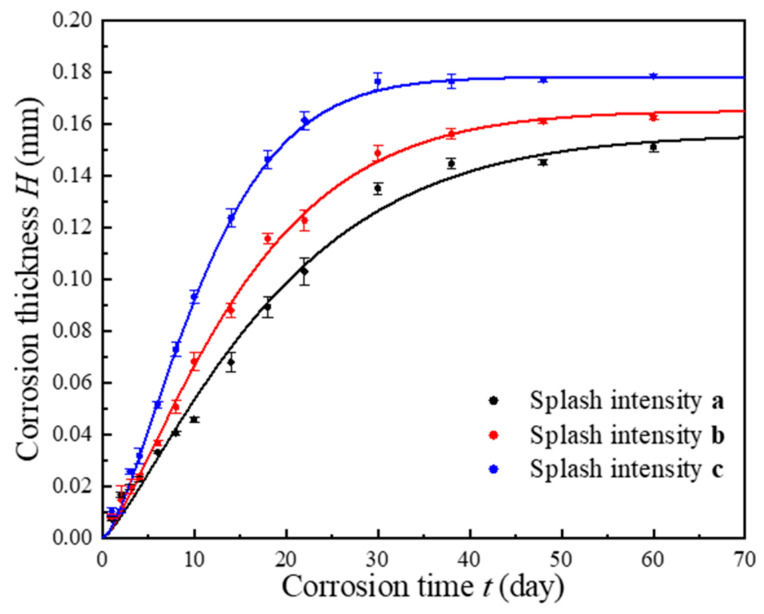
Average corrosion depth fitting graph under different splash intensities.

The ultimate test of the model’s robustness is its ability to accurately fit data from different environments while producing parameters that respond in a physically interpretable manner. The average corrosion depth–time data for the three splash intensities were fitted using the proposed Weibull model. The fitted curves are shown in Figure 14, and the corresponding functions are listed in Table 8. The goodness-of-fit statistics are summarized in Table 9.

The Weibull model achieves consistently high coefficients of determination ($R^{2}$ > 0.99) and favorable results in other statistical tests across all three conditions. This confirms that the model retains excellent descriptive accuracy under varying environmental stress.

Beyond the quality of fit, the systematic response of the fitted parameters to increasing splash intensity reveals their physical consistency:

Maximum Corrosion Depth ($d_{\infty }$): This parameter shows a logical, increasing trend with environmental severity: $d_{\infty }$ = 0.0156 (*a*), 0.0164 (*b*), 0.0178 (*c*). This confirms that $d_{\infty }$ acts as a sensitive indicator of long-term corrosion potential, scaling upward as the environment becomes more aggressive.

Scale Parameter (α): The parameter α decreases from 0.05 (*a*) to 0.06 (*b*) and 0.08 (*c*). A smaller α signifies that the corrosion process reaches its characteristic state more quickly, which is entirely consistent with an increased overall corrosion rate under higher splash intensities. This variation reinforces the physical role of α as a characteristic time constant inversely related to corrosion kinetics.

Shape Parameter (β): Crucially, α remains greater than 1 under all conditions (β = 1.25, 1.29, 1.44 for *a*, *b*, *c*), reaffirming that the “increase-then-decrease” corrosion rate pattern is fundamental to the splash zone environment, regardless of intensity. The gradual increase in β with splash intensity indicates a sharper transition from the rapid to the slow corrosion phase. Physically, this can be interpreted as a more pronounced “delay” in the protective effect of the rust layer under higher splash intensity. More frequent and forceful wetting likely leads to a thicker but initially more porous and less adherent rust layer, allowing the corrosion rate to peak higher and later (reflected in a larger *β*) before the eventual consolidation and stabilization of the layer significantly retards the process. This nuanced response of *β* underscores its sensitivity to environmental kinetics and its value as an indicator of corrosion progression dynamics.

The model parameters do not vary randomly; they respond systematically and in a physically interpretable manner to the controlled environmental variable (splash intensity). This demonstrates that the model’s internal structure is meaningfully coupled to the underlying physico-chemical processes. This parameter consistency is the foundation for the model’s robustness and predictive adaptability to changing field conditions.

The validation conducted in this chapter confirms that the proposed Weibull-based corrosion monitoring model is not a “one-condition” fit but possesses significant environmental adaptability and robustness. Its consistent high accuracy across a spectrum of splash intensities demonstrates reliable performance potential in the face of natural environmental variability. Furthermore, the systematic, physically logical response of its parameters ($d_{\infty }$, α, β) to increased environmental severity provides profound added value. In a deployed monitoring system, tracking the evolution of these fitted parameters over time could yield more than just a corrosion depth readout; for instance, a sustained upward trend in the fitted $d_{\infty }$ could be algorithmically flagged as an indicator of increasing environmental aggressiveness or coating degradation, thereby adding a valuable diagnostic layer to the system’s intelligence.

## 6. Conclusions

This study developed and validated a Weibull distribution-based corrosion depth monitoring model to address the need for intelligent corrosion monitoring of steel structures in marine splash zones. The main conclusions are as follows:

1. A Weibull-based corrosion model suitable for intelligent monitoring was established. The model directly outputs corrosion depth, and its parameters—the maximum corrosion depth ($d_{\infty }$), scale parameter (α), and shape parameter (β)—possess clear physical significance. Designed as the core algorithm for smart sensors, it translates time-series sensor data into quantitative assessments of structural corrosion status and residual life.
2. The model’s accuracy in describing time-variant corrosion was systematically validated. Multi-method analysis integrating corrosion morphology (SEM/EDS), mass loss, and electrochemical impedance spectroscopy (EIS) elucidated a clear three-stage progression (rapid, slow, and stable) for Q355 steel in simulated splash zone conditions. Comparative fitting using mass-loss data demonstrated that the Weibull model is significantly superior to traditional linear and power-law models, achieving a higher goodness-of-fit and accurately capturing the non-linear, phased evolution of corrosion.
3. The model demonstrated robust adaptability to variations in splash intensity. The Weibull model maintained excellent fitting performance across low, medium, and high splash intensities. Crucially, its key parameters responded systematically and with physical consistency to increasing environmental severity: $d_{\infty }$ increased, β remained >1 and showed a slight rising trend, and α decreased correspondingly. This confirms the model’s extrapolative capability and its parameter system’s sensitivity to environmental changes.
4. The model provides an effective tool for intelligent corrosion monitoring and life prediction. The physical interpretability of the parameters enables the model to offer insights beyond mere depth prediction, including diagnosing the corrosion stage and indicating environmental aggressiveness. As such, it can be integrated as the algorithmic core of sensor systems, facilitating the transition from real-time data acquisition to safety assessment and maintenance decision-making, thereby enhancing the lifecycle management of critical marine infrastructure.

Future work should focus on the long-term validation of this model through actual marine exposure trials and further investigation into the coupling effects of multiple environmental factors (e.g., temperature, chloride concentration) on the model parameters to improve its predictive reliability in complex real-world scenarios.

## Figures and Tables

**Table 1:** Chemical Composition of Q355 Steel (Mass Fraction wt, %).

Elements	C	Si	Mn	P	S	Cr	Ni	Cu
Content	0.2	0.55	1.7	0.03	0.02	0.2	0.3	0.3

**Table 2:** EDS analysis results of corrosion products on Q355 steel after various durations of corrosion.

Element (At%)	C	O	Na	Si	Cl	Mn	Fe
3 d	4.16	30.43	-	0.405	0.95	1.45	62.60
8 d	3.85	29.31	-	0.24	3.67	1.03	61.91
22 d	3.34	35.55	0.23	0.17	1.05	0.68	60.75
60 d	5.02	32.03	0.99	-	0.09	0.83	61.05

**Table 3:** Curve-fitting results of the equivalent circuit model for day 1–4.

Corrosion Time	$R_{s}$	$Q_{dl}$		$R_{ct}$	$\chi^{2}$
(Day)	$(\Omega \cdot {cm}^{2}$)	$Y_{dl}(\Omega^{-1}{cm}^{2}s^{n}10^{-6})$	$n_{dl}$	$(\Omega \cdot {cm}^{2}$)	
1	10.79	8089	0.83	161.10	5.22 × 10^−5^
2	8.048	19,660	0.71	94.92	4.63 × 10^−5^
3	31.56	21,010	0.31	35.65	1.83 × 10^−5^
4	9.581	4581	0.62	9.189	2.29 × 10^−5^

**Table 4:** Curve-fitting results of the equivalent circuit model for day 6–22.

Corrosion Time	$R_{s}$	$Q_{dl}$		$R_{ct}$	$Z_{w}$	$\chi^{2}$
(Day)	$(\Omega \cdot {cm}^{2}$)	$Y_{dl}(\Omega^{-1}{cm}^{2}s^{n}10^{-6})$	$n_{dl}$	$(\Omega \cdot {cm}^{2}$)	$Y_{w}(\Omega^{-1}{cm}^{-2}s^{0.5})$	
6	9.042	1500	0.79	25.94	0.01298	1.19 × 10^−4^
8	15.38	32,800	0.71	43.94	0.02145	4.32 × 10^−4^
10	10.25	43,700	0.61	9.1	0.00209	3.06 × 10^−5^
14	13.8	5800	0.63	17.51	0.0241	2.15 × 10^−4^
18	11.69	10,960	0.48	16.71	0.0334	6.39 × 10^−5^
22	8.663	1752	0.75	6.584	0.0311	9.42 × 10^−5^

**Table 5:** Curve-fitting results of the equivalent circuit model for day 30–60.

Corrosion Time	$R_{s}$	$C_{cp}$	$R_{cp}$	$C_{dl}$	$R_{ct}$	$Z_{w}$	$\chi^{2}$
(Day)	$(\Omega \cdot {cm}^{2}$)	${(\mu Fcm}^{2})$	$(\Omega \cdot {cm}^{2}$)	${(\mu Fcm}^{2})$	$(\Omega \cdot {cm}^{2}$)	$Y_{w}(\Omega^{-1}{cm}^{-2}s^{0.5})$	
30	13.88	27.75	1.061	323.7	2.353	0.01685	5.71 × 10^−4^
38	21.98	2.775	4.472	121.5	5.514	0.01683	9.94 × 10^−4^
48	17.86	2.122	2.524	82.46	4.589	0.02398	2.4 × 10^−4^
60	21.1	3.17	4.215	195.6	5.21	0.2716	9.9 × 10^−4^

Note: In the equivalent circuit model for day 30–60 (Table 5), the capacitance of the $C_{cp}$ and the $C_{dl}$ are represented by ideal capacitors for simplicity and because the fitting with CPEs did not yield physically more plausible parameters or significantly improve the $\chi^{2}$ for this stage. The $Y_{w}$ is retained to account for the persistent diffusion process within the thickened rust layer.

**Table 6:** Functional expressions of corrosion models.

Corrosion Model	Functional Expressions
Linear model	$d\left(t\right)=0.00027t+0.0022$
Exponential model	$d\left(t\right)=0.001334t^{0.624}$
Weibull model	$d\left(t\right)=0.0156\times \left\{1-exp\left[-{\left(0.05t\right)}^{1.25}\right]\right\}$

**Table 7:** Summary table of fitting test results.

Models	Linear Model	Exponential Model	Weibull Model
$R^{2}$	0.8889	0.95216	0.9913
χ^2^ test *p* value	0.1873	0.1724	0.6699
χ^2^/DoF	1.7387	1.8616	0.1818
KS test *p* value	0.00096	0.00095	0.00093
KS statistic	0.4987	0.4991	0.4997
AD test *p* value	0.4410	0.6507	0.3172

**Table 8:** Weibull fitting functions for different splash intensities.

Splash Intensity	Functional Expressions
a	$d\left(t\right)=0.0156\times \left\{1-exp\left[-{\left(0.05t\right)}^{1.25}\right]\right\}$
b	$d\left(t\right)=0.0164\times \left\{1-exp\left[-{\left(0.06t\right)}^{1.29}\right]\right\}$
c	$d\left(t\right)=0.0178\times \left\{1-exp\left[-{\left(0.08t\right)}^{1.44}\right]\right\}$

**Table 9:** Summary table of fit test results under different splash intensities.

Splash Intensity	a	b	c
$R^{2}$	0.9913	0.9977	0.9986
χ^2^ test *p* value	0.6699	0.9729	0.2004
χ^2^/DoF	0.1818	0.0011	1.6396
KS test *p* value	0.00093	0.00093	0.00092
KS statistic	0.4997	0.49987	0.49992
AD test *p* value	0.3172	0.37316	0.83431

## Data Availability

The original contributions presented in the study are included in the article, further inquiries can be directed to the corresponding authors.
